# A conceptual matrix model of patient involvement and patient expertise in medicines and medical technology research and development

**DOI:** 10.1186/s40900-026-00926-0

**Published:** 2026-06-20

**Authors:** Adeline Rosenberg, Trishna Bharadia, Guy Yeoman, Liz Clark, Graham R. McClelland

**Affiliations:** 1 Centre for Pharmaceutical Medicine Research, King’s College London, Stamford Street, London, SE1 9NQ UK; 2 Twist Medical LLC, Burlingame, CA USA; 3 The Spark Global, Buckinghamshire, UK; 4 MediPaCe Ltd., London, UK

**Keywords:** Patient involvement, Patient expert, Lived experience, Medicines development, Medical technology

## Abstract

**Background:**

Patient involvement within academic and for-profit medicines and medical technology research and development is happening across organisations and functions. Frameworks exist to conceptualise involvement activities along a spectrum based on patient contributions and their share of voice and decision-making. Examples range from Informing and Education of patients through to Co-design and Co-Production e.g., in research and development. Varying levels of patient expertise profiles also exist based on a spectrum of experience and expertise – from Patient by Experience through to Patient Key Opinion Leader (KOL) – though researchers often lack clarity on the most relevant skills and knowledge of patient partners suited to the task at hand. Here, we propose a conceptual matrix model combining these two concepts into a simple, pragmatic framework intended for patient involvement professionals in research and development, but with broader relevance for other contexts.

**Methods:**

As authors with a mix of academic, for-profit industry, and patient advocacy backgrounds, we utilised intellectual exchange and consensus formation in three workshops to develop the model.

**Results:**

We have developed a conceptual matrix model and applied illustrative case study examples from among our collective experiences and network to demonstrate the model in action. Trends in sample sizes, patient diversity, project longevity, and share of influence exist along the matrix axes. For example, greater numbers and diversity of patients are typically seen at the lower levels of involvement, where there is greater capacity for breadth of involvement.

**Conclusions:**

Our aim for this model is to support and encourage patient involvement professionals to better determine appropriate levels of involvement and patient expertise needed for their projects and activities, enabling them to facilitate more appropriate, fair, effective, and sustainable patient involvement.

## Abstract (plain language summary)

**What is the matrix model?:**

Universities and for-profit organisations regularly involve patients when they research and develop medicines and medical technologies. Patients can be involved in different types of projects. For example, they can review research plans or patient materials. Different patient involvement activities can be thought of in terms of levels on a spectrum. Patient expertise can also be thought of as levels on a spectrum. Some patients may have expertise in their personal experience. Some patients may have more backgrounds and types of expertise, such as in research processes. So far, these two spectra have been used separately. Researchers may not be aware of the different skills and knowledge that patients may have. So, we took these two spectra – the level of patient involvement and the level of patient expertise – and combined them into a single practical tool using a matrix model (graph).

**How did we create it?:**

Our group has a mix of expertise, including university researchers, for-profit industry professionals, and patients. We met over three workshops to develop our matrix. We also chose real-life case study examples of patient involvement activities to show how the matrix can be used.

**How can it be used?:**

The matrix is designed for people working in patient involvement at universities and for-profit organisations to consider what levels of involvement and expertise are best for their projects. It might also be helpful for researchers from other types of organisations. We intend for this to create more useful and effective patient involvement.

## Background

Patient involvement in the research and development of medicines and medical technology is being utilised across research sectors and industry business functions, e.g., research and development, medical affairs, market access, and more [1–3]. Good practices to guide patient involvement professionals working within these contexts have been developed, validated, addressed, and showcased [4, 5]. Equally, there are pitfalls and bad practices to avoid that risk undermining efforts and alienating patient communities, e.g. tokenism [6, 7], though such pitfalls are not always obvious for both those with and without extensive patient involvement experience. Toolkits also abound to guide good patient involvement, but there are arguably too many to choose from, and people who are new to facilitating patient involvement need a simple introductory framework [8].

Patient involvement encompasses a range of contribution levels or input from patients. The lower end of this range, where patients have a lesser share of influence and decision-making, includes activities such as Informing or Educating. The higher end, where patients have a greater share of influence and decision-making, includes activities such as Co-design and Co-production. This concept is demonstrated in the established frameworks of Arnstein’s Ladder of Citizen Participation [9] and Peoplehub’s Spectrum of Participation [10]. Good patient involvement is best communicated through spectra by indicating the relevant level of involvement, and by aiming for as high a level of involvement as is feasible and appropriate for the context. 

At the same time, patient lived experience and expertise also exists on a spectrum, and patients cannot be viewed as a single homogenous group. Examples that demonstrate this include the Pyramid of Patient Types [11] and EUPATI’s Guidance for Patient Involvement in Industry-Led Medicines Research and Development [12]. The lived experience of patients may relate to living with a medical condition, having experience of a treatment modality, or knowledge of a circumstance (e.g., disability or genetic predisposition), depending on the context of the project in which they are involved. Further, patient communities may also have expertise rooted in collectivised knowledge beyond the individual experience [13]. As patients contribute to more patient involvement activities, their skills, experience, expertise, and breadth of knowledge can increase, enabling them to progress from “Patient by Experience” to “Patient Expert” to “Patient Key Opinion Leader (KOL)” [11]. Depending on the context of the involvement activity, it is possible to move between levels and for individuals to represent and relate to multiple patient profiles. When patient involvement activities are in early development, many individuals and organisations who are facilitating involvement may have limited insights along both of these spectra of involvement and patient expertise.

The idea for a matrix combining these two concepts to encourage expansion of understanding evolved out of a research theme from author AR’s doctoral research on patient involvement in publications – which used Peoplehub’s Spectrum of Participation framework – in connection with the Pyramid of Patient Types and additional Centre for Pharmaceutical Medicine Research educational materials [10, 11, 14]. The individual concepts presented here are not new, but they have not been explicitly linked within a simple matrix model, nor brought together as a practical framework for pragmatic application intended for patient involvement professionals in research and development, but with broader relevance for other contexts.

Our aim is to provide an illustrative framework, demonstrated using real-world examples as case studies for patient involvement professionals working within academia and the for-profit medical research industry and adjacent functions, including medicines and medical technology developers, researchers, and regulators. This model will equip individuals and organisations with a better understanding of the distinctions in the different levels of patient involvement and patient expertise profiles to ensure that such activities are optimally set up for success. Additionally, such professionals should be able to better consider how high up the involvement spectrum they can feasibly go based on capacity (i.e., budget, time, and human resource), capability (i.e., skills and competencies), and context (i.e., project objectives and requirements).

The current scope of the conceptual matrix is defined as: patient involvement within academia- or for-profit industry-funded health and medical research and adjacent functions, where individual patients are brought in as external consultants, across the medicines and medical technology research and development lifecycle. Though there is broader relevance to other research sectors and healthcare contexts, for the purposes of development the scope currently excludes patient-led and charity-funded research, as well as the involvement of patient organisation representatives who are contributing on behalf of their organisation. This scope was determined based on our collective experiences and expertise across sectors and contexts, and in recognition of the patient-led research context often representing an inversion of the models and paradigms that informed this work [15], deserving of its own theory and models.

## Methods

Our authorship working group of five individuals represents a mix of multiple perspectives, experiences, and stakeholder backgrounds across academia (all authors), for-profit industry and adjacent functions (authors AR, GY, LC, and GRM), and patient advocacy (authors AR and TB). Our group was formed of a doctoral patient researcher (author AR), a principal investigator (author GRM), a patient engagement research theme lead (author LC) and visiting lecturers in patient engagement (authors TB and GY), all associated with the Centre for Pharmaceutical Medicine Research, King’s College London and participating in this work on the basis of known educational and research contributions to the department on the topic. Further perspectives and expertise represented include a medical communications and patient engagement consultant (author AR), a founder of a patient involvement consultancy (author TB), and a founder of an organisation delivering patient involvement programmes (author GY).

We met over three collaborative workshops in May, August, and November of 2025, each lasting 1–2 h. All workshops were held virtually for accessibility and were facilitated and led by author AR using a Miro online workspace (Miro, San Francisco, CA, USA) for visualisation and participatory interaction [16].

**Fig. 1:**
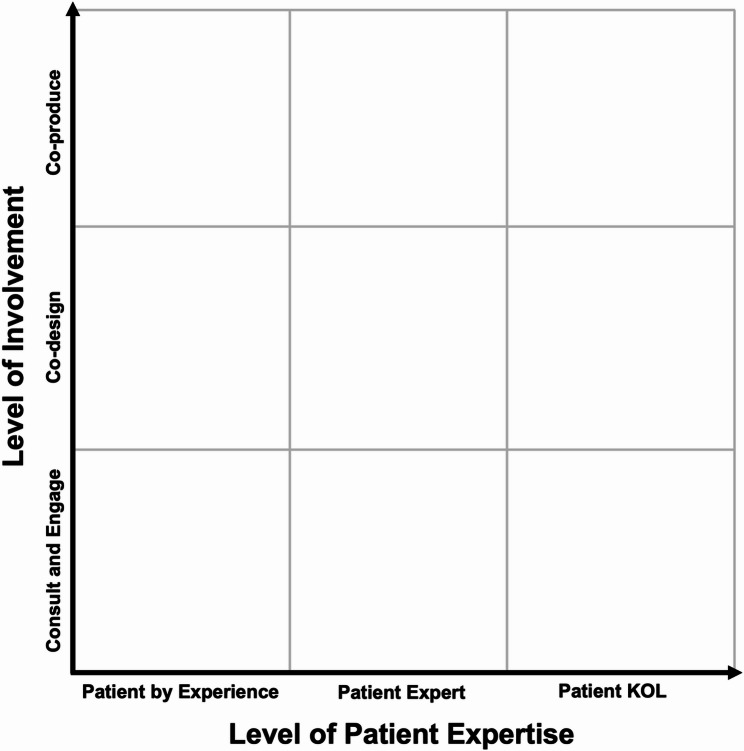
Conceptual matrix of patient involvement. KOL, key opinion leader

**Fig. 2:**
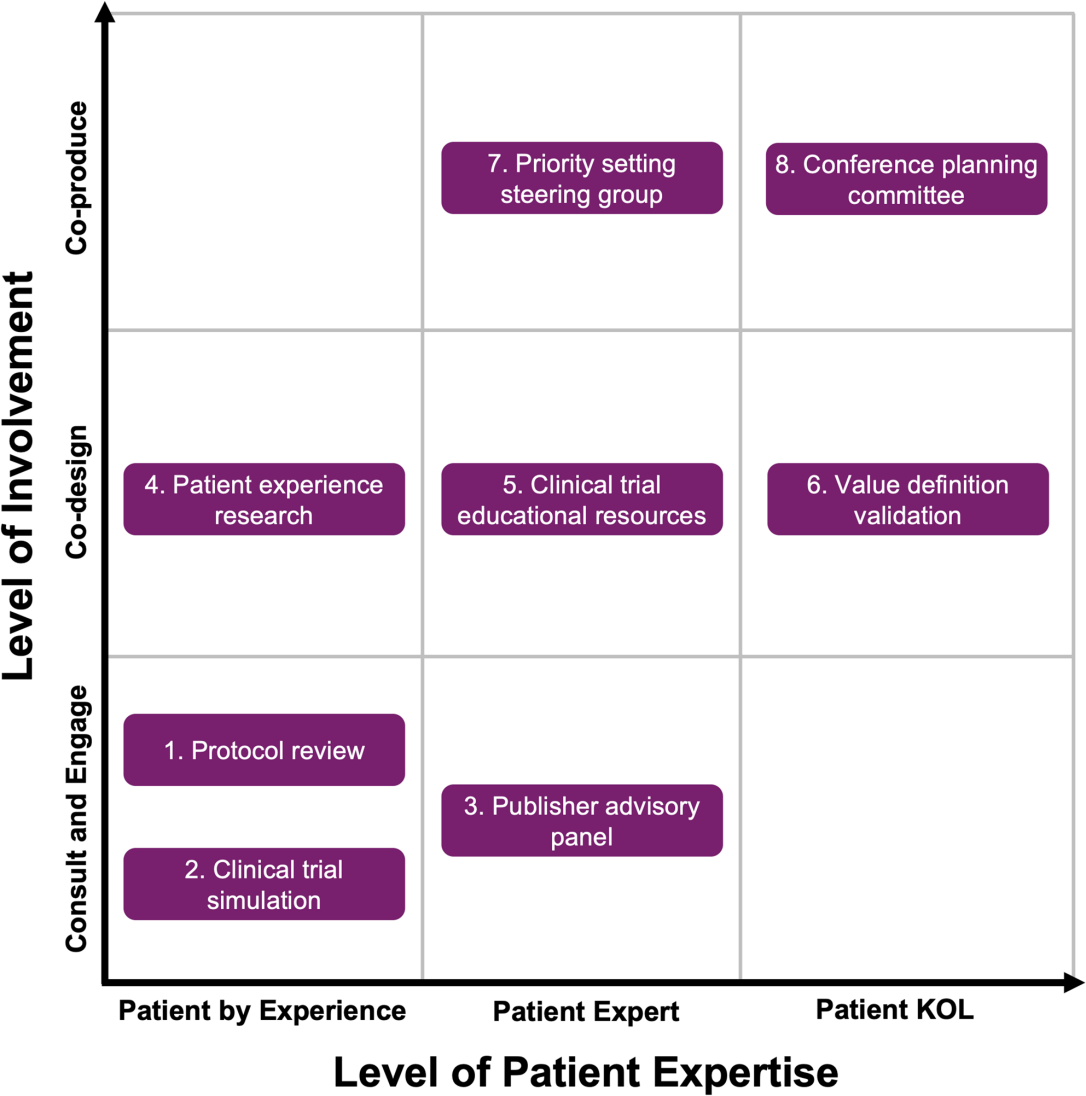
Location of case studies within the conceptual matrix. KOL, key opinion leader

Through discussion, intellectual exchange, and consensus formation, we condensed our materials into a single matrix visual, defined the scope of the model (Workshop 1; see Fig. 1), and aligned on definitions for all levels of involvement and levels of expertise (Workshop 2; see Table 1). We then collected real-world examples from among our collective experiences and outreach to our professional networks to ensure project familiarity and access to firsthand knowledge. Potential case studies were robustly discussed and purposively and subjectively selected to include the most illustrative example for each segment of the matrix. The selection process aimed to ensure a diversity of different types of projects at different stages of medical development activities and from different organisations to demonstrate breadth (Workshops 2 and 3; see Fig. 2). Our criteria for case study inclusion were that they must: adhere to the scope of the matrix; be publicly available and citable; and be able to be clearly located within a single segment of the matrix with minimal overlap to other segments. Case studies were assigned to locations on the matrix based on the levels of involvement and the levels of patient expertise reported. If this information was not available or clear, it was clarified with the author or individual from among our professional networks who had recommended the case study for inclusion.

**Table 1:** Matrix terminology. Definitions for understanding the matrix that were developed and agreed upon during the workshops

Category	Term	Definition
*General Terms*	**Patient**	An individual who lives with or is affected by a disease or condition (i.e. a broad definition of patient that includes those with lived conditions or receiving health or social care, caregivers, and family members) who provides unique and valuable input from the patient perspective. *Source: Adapted from Oliver et al.*, 2022 [29].
	**Patient Involvement**	The active, meaningful, and collaborative interaction between individual patients and organisations across all stages of the medicines and medical technology development lifecycle, where decision-making is impacted by patients’ contributions as partners, recognizing their specific experiences, values, and expertise. *Source: Adapted from Harrington et al. 2020* [1].
	**Organisation**	The industry or industry-adjacent company, regulator, or academic institution that is soliciting patient involvement. *Source: Adapted from workshop discussions.*
*Involvement Level (y axis)*	**Consult and Engage**	Where the organisation is asking patients for their opinions. Consultation typically has more breadth and involves a larger group of patients in closed yes or no decisions, with some opportunity to guide the decision-making. Engagement is a two-way dialogue and feedback loop that typically has more depth and involves a smaller group of patients in open-ended questions to understand their ideas and insights about decisions, with more opportunity to shape decision-making. *Source: Adapted from peopblehub.org* [10].
	**Co-design**	Where the organisation involves a small group of patients in setting objectives, deciding how best to achieve them, and the process of implementation, with good opportunity to influence decision-making. Co-design differs from Co-produce in that it does not include delivery and evaluation. *Source: Adapted from peopblehub.org* [10].
	**Co-produce**	Where the organisation collaborates with a very small and consistent group of patients at a strategic and longitudinal level on the ongoing delivery and evaluation of a project, with ample opportunity to drive decision-making. *Source: Adapted from peopblehub.org* [10].
*Patient Expertise Level (x axis)*	**Patient by Experience**	An individual who has personal experience of a disease or condition (i.e. a broad definition of patient that includes those with lived conditions or receiving health or social care, caregivers, and family members) who provides unique and valuable input from the patient perspective. *Source: Adapted from Oliver et al.*,* 2022* [29].
	**Patient Expert**	A Patient by Experience with expertise in representing their own disease area and knowledge of broader issues impacting their disease community. *Source: Adapted from workshop discussions.*
	**Patient Key Opinion Leader (KOL)**	A Patient Expert with additional expertise in organisational systems, structures, and processes (e.g. regulatory affairs, medical affairs, etc.), combined with leadership and influence within the broader patient involvement community. *Source: Adapted from workshop discussions.*

## Results

Within the matrix (Fig. 1), the y axis – the level of involvement – begins at Consult and Engage. Inform and Educate have been excluded because these stages represent ‘doing to’ and not yet ‘doing with’ patients. Consult and Engage have also been merged into a single level, as although these are presented separately within theoretical frameworks [10], they are often used interchangeably in practice in our collective experience. Table 1 provides definitions of each level of involvement and level of expertise, as well as other terms used in this paper. Figure 2 locates each of the real-world case studies within the matrix. To note, no case studies were identified by the authors for the positions of *Consult and Engage/Patient KOL* and *Co-produce/Patient by Experience.*

Eight case studies are presented below according to each segment of the matrix.

### Consult and Engage/Patient by Experience

As one of the areas with the greatest amount of activity, we have chosen to include two illustrative examples for this segment of the matrix.

1. Protocol review – Pfizer conducted a single face-to-face meeting involving young **Patients by Experience** living with eczema (recruited via the GenerationR Liverpool Young Persons Advisory Group as a gatekeeper organisation) who were **engaged** to review a proposed clinical trial protocol and informed consent documentation. The feedback led to improved consent and assent documentation for the paediatric trial participants [17].
2. Clinical trial simulation – Boehringer Ingelheim conducted a virtual simulation of receiving study information and home spirometry testing for a hypothetical phase 3 trial in pulmonary fibrosis. Eighteen **Patients by Experience** and caregivers participated in the simulation and **consulted** on the information documents and the at-home tasks, leading to adaptations for a more patient-friendly trial protocol [18].

### Consult and Engage/Patient Expert

3. Publisher advisory panel – Future Science Group, now part of Taylor & Francis, established a panel of 26 **Patient Experts** and industry advisors to **consult** on their plain language summary programme and processes and to provide expert peer review. This led to the pioneering of the innovative Plain Language Summary of Publication article type, and after Future Science Group was acquired by the larger publisher, the initiative was scaled up to cover Taylor and Francis’ wider portfolio of journals [19].

### Co-design/Patient by Experience

4. Patient experience research – MediPaCe, with funding from Carl Zeiss Medtech AG, recruited four **Patients by Experience** to a working group to **co-design** and interpret a qualitative interview research study on experiences of radiotherapy in breast cancer. They contributed to refining the research question, outcome measures, and recruitment strategies, and were involved as co-authors on the study results paper [20].

### Co-design/Patient Expert

5. Clinical trial educational resources – This collaboration between the industry organisation PHUSE, the academic institution the Multi-Regional Clinical Trials Centre of Brigham and Women’s Hospital and Harvard, and **Patient Experts** resulted in the co-creation of a series of animations and infographics about clinical trials and data transparency for trial participants and the general public. The patient experts **co-designed** and inputted into the development of the resources, with three out of seven planned videos available on YouTube at the time of the case study article, and complementary infographics in the works [21]. Since the original publication, additional videos and infographics have been made available [22].

### Co-design/Patient KOL

6. Value definition validation – In the second part of a multi-stage project, AstraZeneca hosted two advisory board workshops with 22 **Patient KOLs** and caregivers to share prior survey findings on the defining values of patient centricity for the advisors to **co-design** the values through critique, refinement, validation, and confirmation. This project led to the wider adoption of a patient centricity programme within AstraZeneca [23].

### Co-produce/Patient Expert

7. Priority setting steering group – The Multiple Sclerosis (MS) in the 21st Century steering group project, funded by Merck KGaA (Darmstadt, Germany), is an industry-neutral collaboration between academics, clinicians, patient organisations, and nine **Patient Experts** living with MS to explore real-world data and identify unmet needs. The ongoing project has resulted in the **co-production** of multiple publications, posters, podcasts, and educational support tools for patients [24].

### Co-produce/Patient KOL

8. Conference planning committee participation and chairing – The International Society for Medical Publication Professionals (ISMPP), whose membership largely comprises industry publication professionals and agency communications consultants, has included **Patient KOLs** on the planning committees of its annual conferences, including as co-chairs to **co-produce** educational content. This has led to the prioritisation of patient-centric and patient-driven sessions and presentations, meeting themes, a patient support programme for conference participation, and patient partner meet-ups on the agenda [25].

## Discussion

Our conceptual matrix model combines concepts of the level of patient involvement [10] with the level of patient expertise [11] in the context of medicines and medical technology research development. This work is situated within a broader toolkit of patient and public involvement frameworks and continuums that have been developed for other contexts, for example Arnstein’s Ladder of Citizen Participation [9] Towle’s Spectrum of Involvement in health professional education [26], and the Quebec Model of patient involvement in healthcare and service partnership [27]. These spectra – in particular the level of patient expertise – are dynamic in relation to people, reflecting the often fluid nature of patient involvement [28]; individuals may move up or down in their level of expertise depending on the context and the type of lived experience that is being researched. However, in relation to specific projects, the application of these spectra in identifying the most appropriate patients to involve is static and is a single decision – or series of decisions – made at a given point in time of the project lifecycle.

From conceptualisation to development to pragmatic application of this matrix, we have identified several observations which align with the axes of our matrix and related literature. The numbers of patients involved – and therefore the expected diversity of the patient cohort – is often greater at the *Consult and Engage/Patient by Experience* corner of the matrix [4]. Longevity and continuity of involvement is typically greater at the *Co-produce/Patient KOL* corner [26]. The share of voice, influence, impact, and decision-making as experienced and contributed by patients are also expected to be greater at the *Co-produce/Patient KOL* corner [10].

This contribution to the literature on patient involvement represents a novel synthesis of existing frameworks. In combining the two spectra, we have exposed gaps in practice where the frameworks overlap. Among the real-world case study examples, we had none for the *Co-produce/Patient by Experience* nor the *Consult and Engage/Patient KOL* corners, which might suggest that not all patient expertise profiles are best suited to all levels of involvement. However, it is possible that such involvement work is happening outside of our awareness. It is also possible that if Patient KOLs are first brought in on Consult and Engage activities, they may be able to make a good case for deeper involvement. Further, if these patients are only involved in Consult and Engage activities and are excluded from more useful and effective decision-making and implementation, this could potentially lead to feelings of not being trusted or being true partners in involvement.

Within industry and academia, this model could be used to inform the development of internal processes for patient involvement. As such, the model is intended to be practical and illustrative rather than exhaustive or systematic, balancing the trade-off between simplicity and comprehensive applicability. Limitations therefore arise from utilising our collective networks and experiences for case study identification, which has led to the prioritisation of academic and for-profit industry projects despite broader relevance to other sectors and contexts. However, this same selection process also has strengths in ensuring familiarity, access to in-depth and firsthand knowledge, and a more meaningful understanding for accurate mapping of the case studies against the matrix.

While this model has been developed within the scope of the authors’ collective experiences and expertise (i.e., industry and academia), the concepts and values presented here are broadly applicable for patient involvement in other fields. Future work to broaden and validate this model could involve expansion of the scope to include other research sectors and healthcare contexts, a systematic literature review, and the development of a roadmap for application.

## Conclusions

Ultimately, we hope this model will support and encourage patient involvement professionals to better contextualise their patient involvement work, to better determine appropriate levels of involvement and patient expertise needed for their projects and activities, and to minimise wasted efforts in order to facilitate more useful, effective, efficient, fairer, and sustainable patient involvement.

## Data Availability

No datasets were generated or analysed during the current study.

## Declarations

### Use of generative artificial intelligence

No generative artificial intelligence was used in the development of this manuscript.
